# Dissection of Dynamic Transcriptome Landscape of Leaf, Bract, and Lupulin Gland in Hop (*Humulus lupulus* L.)

**DOI:** 10.3390/ijms21010233

**Published:** 2019-12-29

**Authors:** Ajay Kumar Mishra, Tomáš Kocábek, Vishnu Sukumari Nath, Praveen Awasthi, Ankita Shrestha, Uday Kumar Killi, Jernej Jakse, Josef Patzak, Karel Krofta, Jaroslav Matoušek

**Affiliations:** 1 Biology Centre, Czech Academy of Sciences, Department of Molecular Genetics, Institute of Plant Molecular Biology, Branišovská 31, 370 05 České Budějovice, Czech Republic; ajaymishra24@umbr.cas.cz (A.K.M.); kocabek@umbr.cas.cz (T.K.); sukumari.nath@umbr.cas.cz (V.S.N.); praveen.awasthi@umbr.cas.cz (P.A.); ankita.shrestha@umbr.cas.cz (A.S.); killi@umbr.cas.cz (U.K.K.); 2 Department of Agronomy, Biotechnical Faculty, University of Ljubljana, Jamnikarjeva 101, SI-1000 Ljubljana, Slovenia; Jernej.Jakse@bf.uni-lj.si; 3 Hop Research Institute, Co. Ltd., Kadaňská 2525, 438 46 Žatec, Czech Republic; patzak@chizatec.cz (J.P.); krofta@chizatec.cz (K.K.)

**Keywords:** bitter acids, prenylflavonoids, terpenoids, trichome, *Humulus lupulus*, lupulin glands, RNA sequencing

## Abstract

The hop plant (*Humulus lupulus* L.) produces several valuable secondary metabolites, such as prenylflavonoid, bitter acids, and essential oils. These compounds are biosynthesized in glandular trichomes (lupulin glands) endowed with pharmacological properties and widely implicated in the beer brewing industry. The present study is an attempt to generate exhaustive information of transcriptome dynamics and gene regulatory mechanisms involved in biosynthesis and regulation of these compounds, developmental changes including trichome development at three development stages, namely leaf, bract, and mature lupulin glands. Using high-throughput RNA-Seq technology, a total of 61.13, 50.01, and 20.18 Mb clean reads in the leaf, bract, and lupulin gland libraries, respectively, were obtained and assembled into 43,550 unigenes. The putative functions were assigned to 30,996 transcripts (71.17%) based on basic local alignment search tool similarity searches against public sequence databases, including GO, KEGG, NR, and COG families, which indicated that genes are principally involved in fundamental cellular and molecular functions, and biosynthesis of secondary metabolites. The expression levels of all unigenes were analyzed in leaf, bract, and lupulin glands tissues of hop. The expression profile of transcript encoding enzymes of BCAA metabolism, MEP, and shikimate pathway was most up-regulated in lupulin glands compared with leaves and bracts. Similarly, the expression levels of the transcription factors and structural genes that directly encode enzymes involved in xanthohumol, bitter acids, and terpenoids biosynthesis pathway were found to be significantly enhanced in lupulin glands, suggesting that production of these metabolites increases after the leaf development. In addition, numerous genes involved in primary metabolism, lipid metabolism, photosynthesis, generation of precursor metabolites/energy, protein modification, transporter activity, and cell wall component biogenesis were differentially regulated in three developmental stages, suggesting their involvement in the dynamics of the lupulin gland development. The identification of differentially regulated trichome-related genes provided a new foundation for molecular research on trichome development and differentiation in hop. In conclusion, the reported results provide directions for future functional genomics studies for genetic engineering or molecular breeding for augmentation of secondary metabolite content in hop.

## 1. Introduction

**Figure 1:**
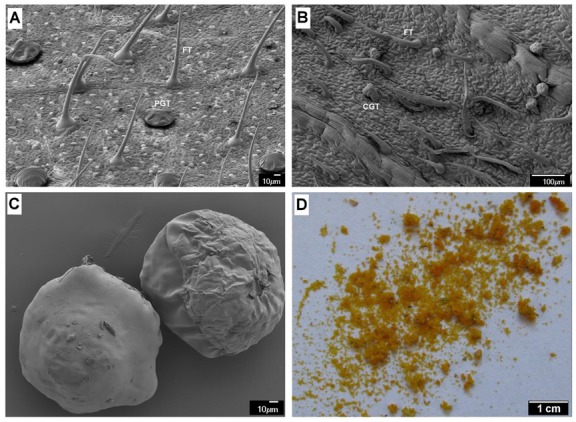
Scanning electron micrograph showing the distribution of different type of trichomes (FT: fibrous trichomes; PGT: peltate glandular trichomes; CGT: capitate glandular trichomes) on the surface of leaf (**A**), bracteole blade (**B**). The scanning electron (**C**) and light microscopy (**D**) image of ripe lupulin glands.

Hop (*Humulus lupulus* L.) is a diploid dioecious perennial climbing plant belongs to the Cannabaceae family, which is cultivated for commercial use primarily in the brewing industry and equally significant to the pharmaceutical industry. The female inflorescences of the hop plants (cones) on the inner side of bracteoles and bracts contain an abundance of metabolically active secretory cells of glandular trichomes known as “lupulin glands” (Figure 1C,D), which biosynthesize and accumulate specialized secondary metabolites such as the prenylated flavonoids (xanthohumol and desmethylxanthohumol), bitter acids (humulone or α-acid and lupulone or β-acid), essential oils, and terpenoids. These compounds are vital ingredients in the brewing industry as a source of bitter flavor, herbal aromas, and natural preservative. Nevertheless, the secondary metabolites of the hop has a long history of utilization for medicinal purposes due to their diverse bioactivities, such as anti-carcinogenic [1], antiinflammatory [2], antimicrobials [3], antioxidant [4], antiglycemic [5], neuroprotective [6], anxiolytic, and sedative effects [7]. In addition to cones, the glandular trichomes are present in lower density on the lower leaf epidermis (Figure 1A), differ morphologically from those present in cones [8], contain low but detectable levels of hop acids, terpenes, xanthohumol and flavonols and serve as the primary site of trichome-specific secondary metabolite biosynthesis [9].

**Figure 2:**
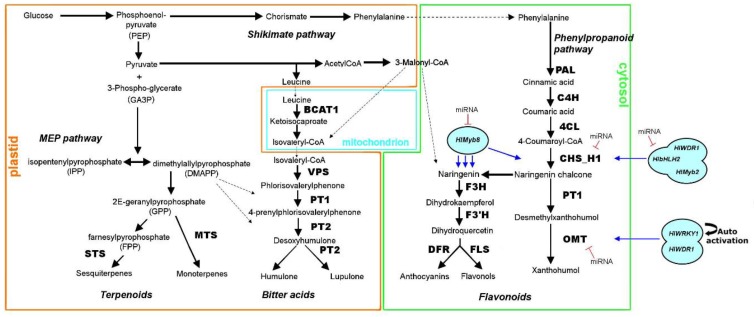
Schematic overview of the biosynthetic pathways (terpenoids, bitter acid, phenylpropanoids and flavonoid) in the hop. The methyl-d-erythritol 4-phosphate (MEP) pathway leading to DMAPP biosynthesis contributes to the bitter acid and terpenoids biosynthesis. Bitter acids are formed from acyl-CoA precursors derived from branched-chain amino acid (BCAA) degradation and MEP pathway. The prenylated chalcones (xanthohumol) biosynthesized via shikimate pathway. The main intermediate compounds are shown with the abbreviation of respective enzymatic steps. Enzyme abbreviations are PAL: phenylalanine ammonia lyase; C4H: cinnamate 4-hydroxylase, 4CL: coumarate coenzyme A ligase, CHS: chalcone synthase, PT: prenyltransferase; OMT: O-methyltransferases; VPS: valerophenone synthase, BCAT: branched chain aminotransferase; F3H: flavanone 3-hydroxylase; F3′H, flavonoid 3′-hydroxylase; DFR, dihydroflavonol 4-reductase; FLS: flavonol synthase; STS: Sesquiterpene synthase; MTS: monoterpene synthases. OMTI and CHS_H1 represent gene isoforms of O-methyltransferases and chalcone synthase genes, respectively in the hop. The movement of metabolic intermediates between cellular compartments is indicated by dashed line.

The development of multicellular glandular trichomes is originated through the enlargement of single epidermal cells, followed by several polarized and localized cell divisions and remodeling to generate branched or unbranched perpendicular structure to the epidermal surface [10]. The glands develop on the abaxial surface of the leaf primordia and continue to form until leaf expansion ceases. During the early stages of the leaf growth, all stages of gland development (pre-secretory, secretory, or post-secretory) can be found on the same blade, although their proportions change, especially before the leaf maturation phase (declination of the pre-secretory glands) [11]. The recent progress in omics and genome editing technologies has generated a genetic framework pointing to the role of transcription factors (TFs), cell cycle regulators, as well as receptors involved in phytohormone-induced signaling cascades in glandular trichome development [12,13]. In Arabidopsis (*Arabidopsis thaliana*), genes including TRANSPARENT TESTA GLABRA1 (TTG1), GLABRA3 (GL3), and ENHANCER OF GLABRA3 (EGL3) forms a regulatory complex and initiate trichome formation by activation of a homeodomain protein GLABRA2 (GL2), which is negatively regulated by single-repeat R3 MYBs [14]. Nevertheless, Arabidopsis contains a single type of unicellular, nonglandular trichome, and our understanding of the molecular mechanism of regulation of glandular trichome development is still relatively limited. Over the past decades, several TF families and genes controlling the glandular trichome formation has been well characterized in other plant species such as a MYB-related TF (MIXTA) from *Antirrhinum majus*; its ectopic expression leads to excess numbers of multicellular glandular trichomes in tobacco [15]; in cucumber, three HD-Zip TFs, namely CsGL1, CsGL2, and CsGL3 are required for glandular trichomes formation [16,17,18]; GoPGF, which encodes a basic helix-loop-helix domain-containing TF is involved as positive regulator of glandular trichome formation in cotton [19]; The Arabidopsis *At*GIS, a C2H2 zinc-finger TF overexpression in tobacco regulates the glandular trichome development through GA signaling pathway [20]. In contrast to other plant species, the molecular mechanisms and regulatory genes which regulate glandular trichome development and density are currently unknown in the hop. Given the way a total of more than 22,000 lupulin gland-specific expressed sequence tags (ESTs) data resource was generated in hop [9], but cell stage-specific, which is essential for advancing our understanding of glandular trichome development lacks in hop. In addition to understanding the molecular genetic basis of lupulin glands development, numerous studies over the last two decades have discovered the role of structural and regulatory genes bitter acids (BA) or prenylflavonoids (PF) biosynthesis in hop. These include the characterization of the role of two aromatic prenyltransferase genes (*Hl*PT1 and *Hl*PT2), which catalyze the major prenylations in the BA pathway in hop [21] (Figure 2). In flavonoid biosynthesis pathway the shikimate pathway serving as a starting point and sequential action of phenylalanine ammonia-lyase (PAL), cinnamate 4-hydroxylase (C4H), and 4-coumaroyl CoA-Ligase (4CL) produce 4-coumaroyl-CoA substrate, which defines a metabolic key branch-point for the synthesis of several major classes of flavonoids, including xanthohumol (PF), flavonol and anthocyanins biosynthesis in hop [9]. The xanthohumol biosynthesis pathway is mediated by trichome-specific chalcone synthase (CHS_H1) [22], *Hl*PT1 [23] and O-methyltransferase 1 (OMT1) [9] enzymes with fine-tuned participation of different types of TFs, belonging to bHLH, MYB, WDR, and WRKY families [24,25,26]. The activation of CHS_H1 promoter is tightly regulated by heterotrimeric ternary MBW (*Hl*Myb3/*Hl*bHLH2/HlWDR1 or *Hl*MYB2/*Hl*bHLH2/*Hl*WDR1) or binary (*Hl*bHLH2/*Hl*WDR1) transcription activation complex [26] (Figure 2). Recently, the transgenic analysis suggested the potential role of other binary transcription activation complex (*Hl*WRKY1/*Hl*WDR1) in positive regulation of MBW ternary complex and OMT1 based on significantly increased levels of PF and BA content in the hop [27]. The biosynthesis of flavanol and anthocyanin is regulated by *Hl*Myb8 TF with the involvement of committed branch point enzyme chalcone isomerase (CHI) [28].

Several works in the last two decades have provided a systematic understanding of secondary metabolite biosynthesis pathway in hop, and despite this progress, a comprehensive characterization of the genes and regulators associated with physiological, morphological transformations and dynamic changes of secondary metabolites in different developmental stages remain poorly understood. The genomic studies have been progressively revolutionized by the high-throughput next-generation sequencing (NGS) technologies. The advancement of RNA-sequencing (RNA-Seq) technology and computational biology tools enables to obtain rapid, and cost-effective transcriptomic resources providing a better insight into transcriptional and post-transcriptional regulation of secondary metabolite biosynthetic pathways associated genes in various non-model plant species such as *Chlorophytum borivilianum* [29], *Camellia sinensis* [21], *Cymbopogon winterianus* [30], and *Polygonum minus* [31]. In hop, it is well documented that lupulin glands in cones contain a significantly high amount of PF and BA compared to leaves and bracts [9], which raises the need to unravel the organ- or tissue-specific expression patterns of related genes of enzymes and regulators.

In this study, first, we performed a comprehensive comparative transcriptome analysis of isolated mature lupulin glands, bracts and leaves of hop and investigated the global expression patterns of genes involved in secondary metabolism biosynthesis, morphological, and developmental differentiation. Subsequently, we investigated the independent metabolomic data for integrated analysis with transcriptome data. Our work will not only provide a step forward approach for unraveling the molecular mechanisms underlying the regulation of gene expression pattern during organ and tissue development factors, which is potentially responsible for the initiation of glandular cells development from the epidermal tissue but also assist in the development of molecular marker and genetic manipulation to improve the quality and quantity of valuable secondary metabolites for brewing and pharmacological sectors.

## 2. Results

### 2.1. Transcriptome Sequence Analysis and De Novo Assembly

**Figure 3:**
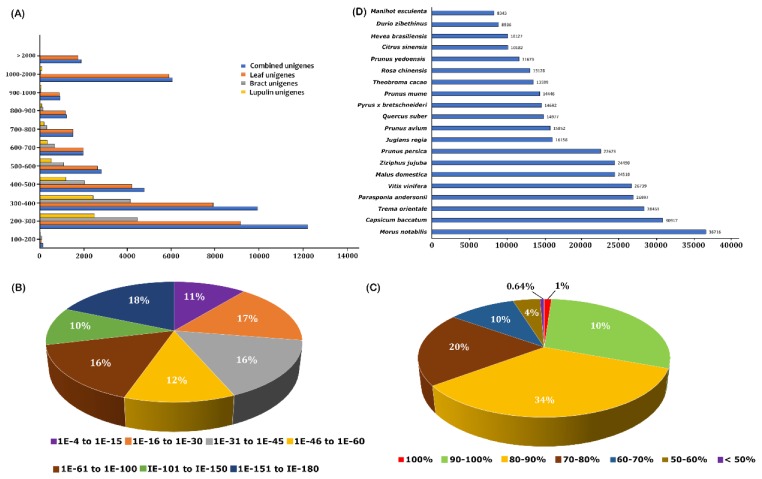
Characteristics of assembled unigenes. (**A**) Length distribution of combined and tissues-specific unigenes; (**B**) E-value distribution of the BLASTX hits against the nr protein database for each combined unigenes (E-value of 1 × 10^5^); (**C**) Similarity distribution of best BLAST hits for each unigenes; (**D**) BLASTx top-hit species distribution of unigenes. All plant proteins in the NCBI nr database were used for homology search and the best hit of each sequence was used for analysis.

To construct a transcriptome database and examine the gene expression profile of hop, a total of nine RNAseq libraries were constructed from leaf (LF), lupulin glands (LG) isolated from mature cones and photosynthetic bracteole and bract devoid of lupulin glands (BR) with three biological replicates and were subjected to Illumina paired-end sequencing. The Illumina Hi-Seq 2500 sequencing platform generated 66.02 Mb, 53.15 Mb, and 22.98 Mb high-quality raw reads for LF, BR, and LG tissues, respectively (Table 1). After the removal of adaptor sequences, low-quality reads (reads with more than 50% of bases with *Q*-value ≤ 20) and trimming sequences consisting of homopolymers more than 8-bp in length, a total of 61.13, 50.01, and 20.18 million high quality filtered reads (Phred quality ≥ Q20) with an average length of 75.14 bp, 108.55 bp, and 112.25 bp were obtained for LF, BR, and LG, respectively (Table 1). The de novo assembly with an optimal K-mer length of 25 and “Reduce” option to reduce redundancy produced 37,690, 13,164, and 7580 unique transcripts (unigenes) for three datasets, corresponding to LF, BR, and LG tissues, respectively, whereas the de novo assembly of all high-quality reads derived from three tissues with the same parameter produced 43,350 unigenes and represents the whole transcriptome. The length-frequency distribution (Figure 3A), N50, N75 value of de novo assembly, average GC content, and the average lengths of unigenes (Table 1), represents the broad view of all three unigene sets and combined transcriptomes. The comparison analysis showed that the N50 and the average length of unigenes derived from three libraries were much longer than those of any specific tissue and previous studies on hop (N50: 650 bp) [32], suggesting the high quality of sequencing data. Pearson’s correlation coefficient between biological replicates were approximately 0.9, suggesting the high reproducibility across samples and credibility of RNA-seq results (Supplementary Materials Figure S1).

### 2.2. Functional Annotation and Classification of Assembled Transcriptome

To understand the putative gene functions of hop transcriptomes in the context of systems biology, all assembled unigenes were subjected to annotation using BLAST searches against several public databases. The BLASTx annotation results showed that 30,996 (71.17%) unigenes had significant similarity with protein sequences in the nr database at E-value cut-off of 1 × 10^5^, whereas, small proportion of sequences (12,554 unigenes, 28.83%) was unannotated and did not exhibit significant homology with sequences in the nr database, which might represent novel, non-protein-coding hop specific transcripts or could be derived from 3′ or 5′ untranslated regions (UTR) of the less conserved genes. The E-value distribution of unigenes annotated to the nr database showed that approximately 10.89% of unigenes mapped in the range of 1 × 10^5^ to 1 × 10^100^ (Figure 3B). The similarity distribution illustrated that the majority of unigenes (99.36%) had homology between 50% and 100%, whereas only 0.64% of the unigenes with homologous sequences had similarities less than 50% (Figure 3C). These results reflected the high homology match of the mapped sequences with known sequences, suggesting the high quality of assembled transcripts. Furthermore, sequence homologies of assembled unigenes showed that 36,716 of total unigenes were matched to sequence from *Morus notabilis* (84.30%), followed by *Capsicum baccatum* (70.99%), *Trema orientale* (65.33%), *Parasponia andersonii* (61.99%), and *Vitis vinifera* (61.39%) (Figure 3D).

**Figure 4:**
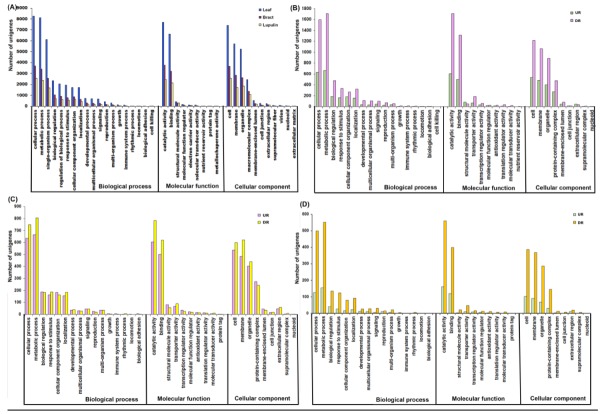
Comparison of Gene Ontology (GO) classifications of combined and tissues-specific unigenes (**A**) differentially expressed genes in bract compared to leaf (**B**), lupulin glands compared to bract (**C**) and lupulin glands compared to leaf (**D**). The results are summarized in three main categories: Biological process, Cellular component, and Molecular function. The *x*-axis indicates subcategories; right *y*-axis indicates number of genes in a category. UR: up-regulated, DR: down-regulated.

In terms of completeness, BUSCO analysis against a core set of 1440 single-copy orthologous genes of plants indicated the presence of 72% as complete, 17.5% fragmented genes, with only a minor fraction of missing orthologs (10.5%) in our de novo assembly, suggesting the high accuracy of assembled transcript sequences. The functional classification of unigenes based on BLAST search against the gene products in the Gene Ontology (GO) database categorized 22,081 unigenes into 39 functional groups under the three main categories namely, biological processes (19 sub-groups), cellular components (11 sub-groups), and molecular functions (9 sub-groups), with multiple terms assigned to the same unigenes (Figure 4A). Under the biological processes category, the most abundant groups of transcript sequences were ‘metabolic process’ (12,280), ‘cellular process’ (11,788) and ‘biological regulation’ (3609) were prominently represented. Among the category of molecular function, the highest proportion of genes were clustered into ‘catalytic activity’ (12,367), ‘binding’ (10,733), and ‘transporter activity’ (1298). Within the cellular components, most transcripts were assigned to ‘cell’ (8790), ‘membrane’ (8505), and ‘organelle’ (6637).

The Clusters of Orthologous Groups (COG) database was used for comprehensive evolutionary functional classification of hop transcriptome. Overall, 19,760 (45.37%) unigenes showed significant homology in the COG database (Figure S2) with several functions resulting in 20,667 functional annotations and these unigenes were classified into 25 COG functional categories. Among these COG categories, the cluster of “function unknown” (5703, 28.86%) represented the largest group, followed by “Signal transduction mechanisms” (2105, 10.7%), “Posttranslational modification, protein turnover, chaperones” (1807, 9.2%), “Transcription” (1457, 7.4%) and “Carbohydrate transport and metabolism” (1234, 6.3%). The assigned functions of unigenes covered a comprehensive range of GO and COG functional classification, suggesting that the assembled unigenes represented a wide diversity of transcripts in hop genome.

In addition, hop transcriptome and unigenes of different tissues were mapped to the reference canonical pathways in the Kyoto Encyclopedia of Genes and Genomes (KEGG) pathway database for a systematic understanding of their complex biological functions and involvement of specific unigenes into biosynthetic pathways in hop. KEGG pathway mapping assigned 14,364 hop transcriptomes (32.98% of all unigenes), 8258 (18.96%) LF, 3134 (23.80%) BR, and 2284 (30.13%) LG unigenes to five main categories: “metabolism”, “genetic information processing”, “environmental information processing”, “cellular process”, and “organismal systems”, with 43 sub-categories and 150 KEGG pathways (Table 2). In all tissues, metabolic pathways constituted the largest category (LF: 1268; BR: 451; LG: 443) followed by biosynthesis of secondary metabolites (LF: 612; BR: 333; LG: 214) including PF biosynthesis, BA biosynthesis, sesquiterpenoid, and triterpenoid biosynthesis, terpenoid backbone biosynthesis, carotenoid biosynthesis, and so on.

Notably, KEGG pathway-based analysis revealed that a total of 88 transcript sequences were associated with the phenylpropanoid biosynthesis pathway, including PF (12 transcripts), flavonoid (43 transcripts), and flavone and flavonol (6 transcripts) biosynthesis pathways. Two hundred and eighty-six transcripts representing the terpenoid biosynthesis, including the synthesis of the tetracyclic diterpenoid backbone (80 transcripts), monoterpenoid (13 transcripts), diterpenoids (23 transcripts), and sesquiterpenoid and triterpenoid biosynthesis (39 transcripts) were identified. In addition, a total of 21 transcripts encode sequences representing the branched-chain amino acids (BCAA) biosynthetic pathway-related candidate genes were identified for the BA biosynthesis pathway. The candidate transcripts with significant matches to BCAA, methyl-D-erythritol 4-phosphate (MEP) and shikimate pathway genes were more annotated in LG than LF and BR, suggesting that levels of PF, BA, and terpenoids increase during the glandular trichome development. The pathway ko00942, which represents the anthocyanin biosynthesis pathway, LF tissues have four genes annotated than in BR (3) and LG (1) tissues. Overall, identification and characterization of candidate transcripts associated with specific pathways will notably advance the understanding of their putative functions during hop development process.

### 2.3. Analysis of Expression Profiles of the Unigenes in Different Tissues

**Figure 5:**
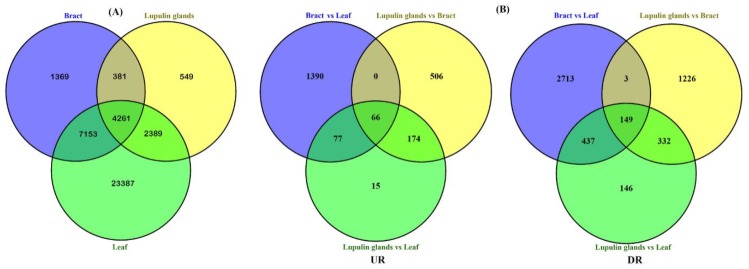
Venn diagram representing the number of unigenes in leaf, bract, and lupulin glands (**A**) and overlap of differentially expressed genes in the pairwise comparison sets of the three stages (**B**). UR: up-regulated, DR: down-regulated.

The expression patterns of each transcript in LF, BR, and LG tissues were digitally measured, and the transcripts with significant differential expression level (logFC ≥ 2 or ≤−2 with *p*-value  ≤  0.05) were characterized based on their FPKM values. We found that, of the 43,550 unigenes, 4261 (9.8%) expressed in all tissues, whereas, 23,387 (53.70%) unigenes were exclusively expressed in LF tissues (Figure 5A), indicating the spatially regulated process of selectively expressed specific fractions of the same genome in LG tissues. Comparative transcript abundance level revealed that 1532 unigenes were up-regulated and 3302 unigenes were down-regulated in BR compared to LF tissues (Table S1), whereas 746 up-regulated and 1710 down-regulated unigenes were obtained in LG vs. BR comparison (Table S2). The number of DEGs was highest between the BR vs. LF and lowest between the LG vs. LF (Table S3). A minor fraction of common genes (215) were found to be differentially regulated in all the comparisons of stages with each other (Figure 5B), showing discordant expression patterns of unigenes. Among the identified DEGs, secondary metabolite biosynthesis pathway-related genes exhibited a greater extent of transcriptional variations at the LG stages (Tables S1–S3).

**Figure 6:**
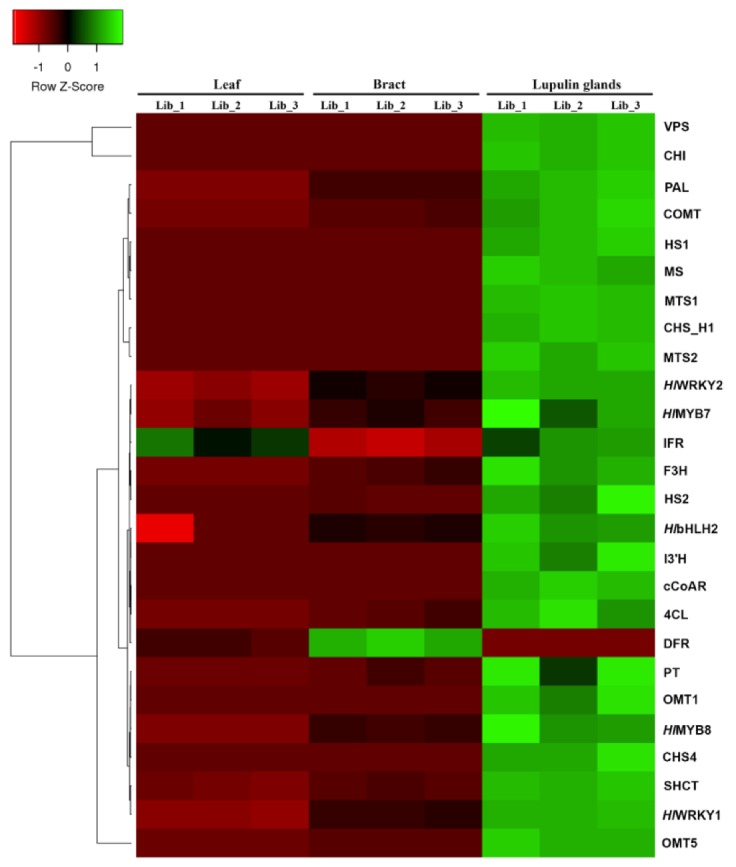
Heat map and complete linkage hierarchical clustering analysis of relative expression levels of differentially expressed secondary metabolite genes involved in important secondary metabolite biosynthesis. Colors on vertical represent the clustered genes based on gene expression, the horizontal line represents the single gene and color of the line indicates the average gene expression in libraries of leaf, bract, and lupulin glands. The signal ratios were shown in a black-green color scale, where green indicated high expression level and black indicated low expression level. COMT: caffeic acid 3-o-methyltransferase; 4CL: 4-coumarate:CoA ligase; cCoAR: cinnamoyl-CoA reductase; MTS2: myrcene synthase; HS2: humulone synthase 2; CHI: chalcone isomerase; VPS: valerophenone synthase; CHS_H1: chalcone synthase; PAL: phenylalanine ammonia-lyase; MTS1: monoterpene synthase 1; SHCT: shikimate o-hydroxycinnamoyltransferase; OMT5: O-methyltransferase 5; I3′H: isoflavone 3′-hydroxylase; OMT1: O-methyltransferase 1; MS: myrcene synthase; HS1: humulone synthase 1; CHS4: chalcone synthase 4; F3H: flavonol synthase/flavanone 3-hydroxylase; DFR: dihydroflavonol 4-reductase; IFR: isoflavone reductase-like protein; PT: prenyltransferase. The color scale (−2 to 2) is shown on the left; red represents low content, green represents high content.

To further investigate gene expression patterns in a pairwise comparison of three stages, we performed hierarchical clustering of selected secondary metabolite related DEGs based on FPKM values using the Euclidean distance method and complete linkage. The hierarchical clustering revealed that the expression levels of most DEGs were almost similar among the biological replicates in each stage (Figure 6), reflecting that the overall transcriptional differences are subtle. The majority of secondary metabolite related genes except dihydroflavonol 4-reductase (DFR) exhibited enhanced expression patterns in LG compared to LF, and BR tissues (Figure 6).

The organ-specific differential gene expression reflects the relevant biological function to that organ. Thus, we performed the GO assignment to classify DEG functions and further investigated the GO term over-representation of DEGs in a pairwise comparison of three stages. The GO annotation categorized 4111 DEGs in BR compared to LF (Figure 4B), 2065 DEGs in LG compared to BR (Figure 4C), 1195 DEGs in LG (Figure 4D), compared to LF into 56 subcategories, within three main categories molecular functions, biological processes, and cellular components, whereas, remaining DEGs (BR vs. LF: 896; LG vs. BR: 434; LG vs. LF: 201) were not classified. In the biological processes category, “metabolic process,” followed by “cellular process” and “biological regulation” were found to be the most represented subcategories in all pairwise comparison of three stages. As for molecular function, the major subcategories were “binding” and “catalytic activity”, whereas, in the cellular components category, “cell” and “membrane” comprised the largest proportion. The GO enrichment analysis showed that sugar and carbohydrate, fatty and amino acid processes were enriched in LF and BR compared to LG (Tables S4–S6; Figures S3–S5), suggesting that they served as a resource to supply of biosynthetic precursors for secondary metabolite biosynthesis in LG tissues. Intriguingly, GO terms such as “secondary metabolite biosynthesis”, “oxidoreductase activity”, “transporter activity” were significantly enriched in LG compared to LF and BR tissues, suggested the high commitment of LG tissues for secondary metabolite biosynthesis. The interactions between the cellular redox signaling hub and the phytohormone signaling network have been reported to play an essential role in plant growth and development as well as biosynthesis of biological compounds [33,34]. The enrichment of “oxidoreductase activity” GO terms underpin the critical role of oxidative signals or cycles of oxidation and reduction in secondary metabolite biosynthesis as well as the development and differentiation of LG tissues.

KEGG annotated analysis were performed for DEGs in the BR vs. LF, LG vs. BR and LG vs. LF stages comparison (Table 2) and in the BR vs. LF stage comparison, 2172 DEGs were annotated; of these 656 up-regulated unigenes were assigned to 168 pathways, and 1516 down-regulated unigenes were assigned to 198 pathways. In the BR vs. LG stage comparison, 1028 DEGs were annotated in the KEGG pathway classification, among them 331 up-regulated unigenes were assigned to 142 pathways, and 697 were assigned to 160 pathways. In the LG vs. LF stage comparison, 586 DEGs were annotated to the KEGG pathway, of these 331 up-regulated unigenes were assigned to 81 pathways, whereas 1039 down-regulated unigenes were assigned to 148 pathways. Notably, according to the KEGG annotation, six genes related to sesquiterpenoid and triterpenoid biosynthesis, four genes related to flavonoid biosynthesis pathway, six cytochrome P450 monooxygenases genes, four genes related to TCA cycle (malate dehydrogenase, isocitrate dehydrogenase, ATP citrate (pro-S)-lyase, aconitate hydratase), four genes related to MEP pathway, four genes related to sucrose metabolism were most predominantly expressed in LG compared to other tissues.

**Figure 7:**
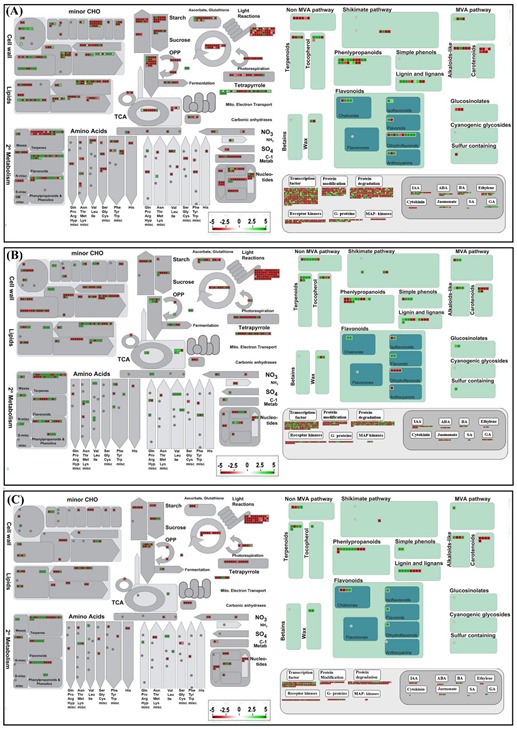
Overview of the MapMan visualization of differentially expressed genes in three stages. (**A**) bract compared to leaf, (**B**) lupulin glands compared to bract, and (**C**) lupulin glands compared to leaf. The log_2_ fold changes of significantly differentially expressed genes were imported and visualized in MapMan. Red and green displayed signals represent a decrease and an increase in transcript abundance, respectively in the pairwise comparison sets of the three stages. The scale used for coloration of the signals (log_2_ ratios) is presented.

Furthermore, DEGs were imported into MapMan for pathway-based analysis and visualization to gain an unbiased systematic overview of the involvement of DEGs in biological processes and cell functions. The DEGs associated with MVA, BCAA, shikimate pathway, and secondary metabolism pathways were enriched, whereas photorespiration and photosynthesis-related DEGs were diminished in LG as compared to LF and BR tissues (Figure 7). Furthermore, the categories that showed the most significant changes included CHO metabolism, hormone signaling, TFs, ubiquitination, signaling G-proteins, receptor kinases indicate the intricate nexus of molecular mechanism participate/changes in the BR and LG development as well as secondary metabolite production in hop.

### 2.4. Validation of the Candidate Gene Expression Patterns by qRT-PCR Analysis

**Figure 8:**
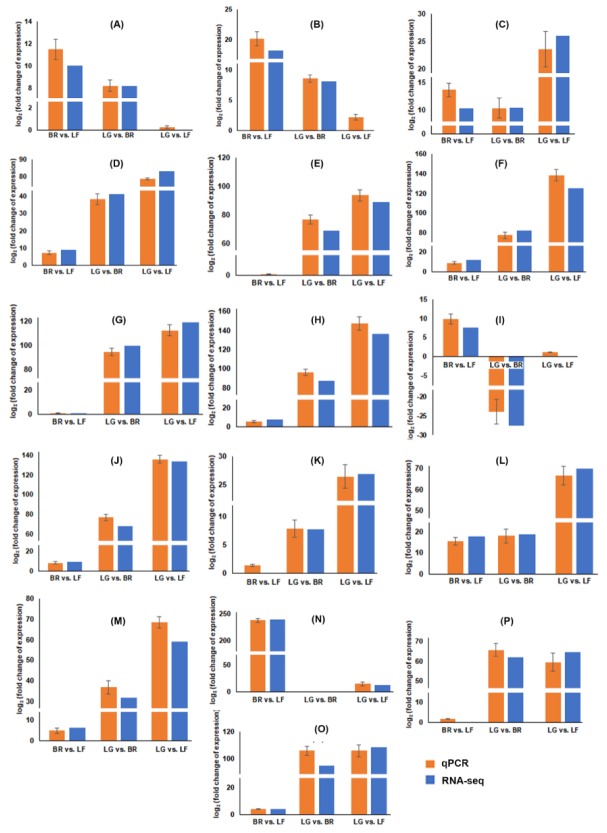
Validation of expression patterns of differentially expressed genes by RT-qPCR. Graph showing fold change of the structural genes, regulatory genes and two randomly selected genes in the pairwise comparison sets of the three stages, namely, bract compared to leaf (BR vs. LF), lupulin glands compared to bract (LG vs. BR) and lupulin glands compared to leaf (LG vs. LF). (**A**) PAL: phenylalanine ammonia lyase; (**B**) 4CL: coumarate coenzyme A ligase; (**C**) C4H: cinnamate 4-hydroxylase; (**D**) CHS_H1: chalcone synthase isoform 1; (**E**) OMT1: O-methyltransferases isoform 1; (**F**) PT1: prenyltransferase 1; (**G**) VPS: valerophenone synthase; (**H**) CHS4: chalcone synthase isoform 4; (**I**) DFR: dihydroflavanol reductase; (**J**) CHI: chalcone isomerase; (**K**) bHLH2 transcription factor; (**L**) *Hl*WRKY1 transcription factor; (**M**) *Hl*MYB8 transcription factor; (**N**) ERT: ethylene-responsive transcription factor ERF017; (**O**) SHCT: shikimate o-hydroxycinnamoyltransferase; (**P**) *Hl*MYB8 transcription factor. qRT-PCR analyses were normalized using DEAD-box ATPase-RNA-helicase (DRH) as an internal control gene. The fold change of each gene was calculated by the 2^−ΔΔCT^ method. Data were means ± standard deviations (SD) of three biological replicates.

To confirm the accuracy of the Illumina paired-end sequencing and FPKM calculated results, the expression profiles of selective structural and regulatory genes cognate to PF, BA, and flavonol biosynthesis pathways and candidate DEGs with high or low expression levels in LF, BR, and LG tissues was validated by quantitative real-time PCR (qRT-PCR). The real-time gene expression analysis suggested that selected structural (except DFR) and regulatory genes were highly expressed in LG compared to BR and LG (Figure 8). The expression of VPS exhibited the LG-specific pattern, with a 100-fold increased expression compared to that in BR. The qRT-PCR results of highly up-regulated and down-regulated genes were in good correlation with those obtained by transcriptomic data. Overall, the consistency of trends between qRT-PCR and transcriptome data provided a credible reference for further studies.

### 2.5. Metabolite Profiling of Important Secondary Metabolite Content

The distribution of important secondary metabolites in LF, BR, and LG tissues of hop was quantified using HPLC (Table 3). The cohumulone, colupulone, and desmethylxanthohumol (DMX) were not detected in LF tissues. The bitter acids and their derivatives (cohumulone, colupulone) started accumulating in BR tissues and reached their highest levels in LG tissues, which is correlated well with higher expression of BCAA genes in LG tissues. The PF such as DMX, and xanthohumol (XN) was elevated in significant amount in LG tissues, suggesting that the LG is the primary site of their accumulation.

## 3. Discussion

The importance of hop in the brewing industry has been undisputed over the centuries with long-history of traditional medicinal implication in Europe. The previous comparative transcriptome analysis of hop tissues was mainly focused on the differential expression of genes responsible for the tissue-specific accumulation of bitter acid [35]. In the present study, we performed the transcription profiles of two organs capable of glandular trichomes production and mature isolated lupulin glands to gain an overview of the genetic composition of LF, BR, and LG tissues; molecular changes that occur during three developmental changes, regulator genes for LG development, and molecular regulation of the biosynthesis of all classes of important secondary metabolites in hop.

### 3.1. Building a Transcriptome Resource and Gene Annotation

In spite of the availability of reference genome, the de novo transcriptome assembly provides an effective means to reconstruct the complete set of transcripts, quantitative assessment of gene expression for specific tissues and identification of novel transcripts, which are often missed in genome assembly process or due to undefined annotation [36]. In the present study, a total of 128 million clean reads were obtained after trimming and performing error corrections from the three distinct tissues, representing 12.94 Gb sequences were de novo assembled into a total of 43,550 unigenes (Table 1). The number of transcripts and an average N50 length of assembled data was slightly higher than RNA-Seq reports of hop genome [32], indicating that sequencing data including transcriptomes were of high quality and integrity and could serve as a basis for functional gene studies and marker development in hop.

Functional classification annotated more than 71.17% (30,996) of unigenes to at least one putative function by known proteins from Nr, COG, KEGG, GO, and other databases, whereas, remaining 28.83% of the assembled unigenes were found to be without any functional annotation, which could be attributed to either the absence of homologous sequence matches in the database or unigenes matched protein with unknown functions. These unigenes were considered as novel transcripts and could serve as valuable resources for further research.

The degradation of the BCAA such as leucine, valine, and isoleucine, generates the prerequisite element iso-valeryl-Co as one of the precursors, which along with two other precursors, namely, malonyl-CoA (polyketide extender) and dimethylallyl diphosphate (DMAPP)serve as prenyl donor involve in BA (α- and β-acid) biosynthesis in hop [37]. The polyketide core of BAs is formed by the condensation of BCAA-derived acyl-CoA with three molecules of malonyl-CoA catalyzed by VPS [37]. The enzymatic step of BCAA biosynthesis generates leucine and valine from pyruvate [38], whereas DMAPP involved in the prenylation of BAs and synthesis of terpenoids is generated by the MEP pathway [23]. Based on our transcriptome sequencing, we identified 14 unigenes involved in the MEV pathway, 4 transcripts with significant matches to terpene synthase (TPS), and triterpene synthase (TTS) genes, and 8 transcripts representing putative Cytochrome P450 monooxygenases (CYP450s) and glycosyltransferase (GT). CYP450s and GT modify the triterpene carbon ring and play essential roles in terpenoids and alkaloids biosynthetic pathways in plants, thus provide an attractive component for large-scale production of terpenoids via metabolic engineering [39,40]. The role of CYP450s and GT has not been elucidated and could subject to future studies in terpenoid biosynthesis in hop. Intriguingly, the essential genes involved in BA biosynthesis such as VPS and branched-chain aminotransferase (BCAT) enzymes such as *Hl*BCAT1 and *Hl*BCAT2, which play critical roles in the formation of branched-chain acyl-CoAs were found exclusively in BR and LG, reinforcing the previous notation that BAs are synthesized in LG [9,35]. In a way similar to BA and terpenoids biosynthesis, 4CL generated from phenylpropanoid biosynthesis pathway condensed with three molecules of malonyl-CoA with the aid of enzyme CHS_H1 to yield the aromatic core of PF [25]. RNA-seq analysis revealed that TFs such as *Hl*bHLH2, *Hl*WDR1, *Hl*WRKY1, *Hl*MYB1, *Hl*MYB2, *Hl*MYB3, *Hl*MYB7, and *Hl*MYB8 [25,26,28] essential for regulating PF biosynthesis were present in LF, BR, and LG transcriptomes, which was in agreement with previous studies that LF contain low but detectable level of PF [9]. Nevertheless, 4.7% (2.076) of the assembled transcripts, 5.2% (1975) LF transcripts, 4.8% (637) BR transcripts, 4.9% (375) LG transcripts were annotated to encode for TFs, which could be classified into 93 TF families, including MYB, bZIP, WRKY, C2H2, and bHLH families. The role of several LG gland specific putative TFs have not been documented and their further functional characterization could mine important regulatory genes for secondary metabolite production in hop.

### 3.2. The Differential Expression Patterns of Unigenes in Different Tissues

The differential gene expression patterns were systematically performed to investigate the active biological pathways in three different hop tissues. The comparison of gene expression analysis showed that key regulatory genes involved in secondary metabolite biosynthesis were significantly upregulated in LG in comparison to LF and BR, underpinning the predominant involvement of LG tissues in specialized biosynthetic pathways than other two organs. The shikimate pathway provides carbon skeletons for the phenylpropanoid pathway, and PAL is the first committed enzyme in the flavonoid metabolic pathway, which controls the metabolic flux towards phenylpropanoid compounds [41]. The elevated levels of PAL and CHS_H1 in BR and LG compared to LF suggested that redirection of carbon flux toward flavonoid biosynthesis defines the size of the PF, flavonols, and anthocyanin in BR and LG tissues in hop. The branch point metabolite naringenin chalcone, which colimits the flux between PF and flavanols/anthocyanin biosynthetic products is regulated by the downstream enzyme activity and TFs [9,25,26]. The higher transcript levels of OMT, *Hl*PT1 and core TFs of MBW and WW complexes in LG compared to BR and LF suggested thatthe major metabolic flux shift of primary metabolism was towards PF biosynthesis in LG tissues. The regulatory input of *Hl*Myb8 and enzyme activity of flavonol synthase (FLS) and DFR define the coarse control of flavanols and anthocyanin biosynthesis pathways, respectively [28]. The elevated expression of *Hl*Myb8, FLS, and down-regulation of DFR, UDP glucose, Flavonoid-3-o-glucosyltransferase (UFGT6), 3-GT, leucoanthocyanidin reductase (LAR), and leucoanthocyanidin dioxygenase (LDOX) enzyme activity may explain the significantly lower anthocyanin content in LG [42]. The present study shows the pronounced expression of genes related to MEP and BCAA pathways and LG specific expression of VPS, terpenoid-related synthases such as sesquiterpene synthase (STS1), monoterpene synthases (MTS1 and MTS2) genes suggested that secretory tissues synthesizing large amounts of terpenoids and BA, which was in agreement with earlier similar findings [9,35].

The transcriptional activation of genes encoding enzymes and TFs of secondary metabolism can efficiently reprogram primary metabolism, driving carbon flux, stored energy, and reducing power in the form of ATP and NADPH/NADHgenerated through central metabolism toward aromatic acid biosynthesis [43]. The increased content of aromatic amino acids and sink strength of the primary metabolism results in decrease content of the major sugars in LG (Figure 7) revealed its dependency on surrounding green tissues for the supply of sugars. Nevertheless, enhanced activity of sugar transporter genes such as sugar transport 13, SWEET family of sugar transporters, ATP binding cassette (ABC) transporters, plastidic ATP/ADP-transporter underscoring the exogenous supply of sugars and energy to LG tissues, corroborating the previous finding that sucrose serves as an excellent and ideal source for secondary metabolite production in glandular trichomes [44]. The gene family (*tropinone reductases*) regulating the tropane alkaloid biosynthesis [45] were notably highly expressed in LG compared to BR and LF, which indeed was inconsistent with the previous result which demonstrated the absence of alkaloids in hop extracts [46]. In this context, the candidate substrate or function of tropinone reductases can open novel directions for future investigation in hop. In discrete phyla and/or tissues of higher plant seeds, acyl-ACP protein thioesterases (acyl-ACP TEs) act as a biochemical determinant of the fatty acid compositions and essential oil biosynthesis [47]. The up-regulated expression of two acyl-ACP TEs genes in LG compared to LF and BR suggested their plausible involvement in glandular trichomes-specific biosynthesis of essential oils in hop.

The molecular studies provide growing evidence to genes playing a specific role in glandular trichome development, including TFs, cell cycle regulators, as well as phytohormone biosynthesis [48]. Intriguingly, we noticed that several TFs, which have been reported to play crucial roles in trichome development were up-regulated in LG tissues. Among others, the negative regulator of trichome development ETC1 encoding single-repeat R3 MYB TF was predominantly up-regulated in LF and BR tissues in comparison to LG tissues. The previous study demonstrated that ETC1 and ETC3 participate in controlling trichome formation on inflorescence stems and pedicles [49], suggesting these genes as essential regulators responsible for glandular trichome density formation in developing cones. The other imperative TF involved in trichome differentiation is CAPRICE (CPC), which was found to be down-regulated in LG in comparison to LF and BR. Loss-of-function in CPC causes increased trichome density [50], its down-regulation suggesting a role in trichome density formation in BR tissues. It is worth noting that another trichome development-related gene encoding for homeodomain-leucine zipper TFs ATHB-51 was up-regulated in LG. The previous study demonstrated that ATHB-51 interacts with meristem regulator LEAFY and participates in trichome formation, floral meristem determinacy, and leaf morphogenesis in *Arabidopsis* [51] and was found to be an essential regulator of multicellular trichome development in *Cucumis sativum* [52]. The expression of the MIXTA, a MYB TF that is considered as a positive regulator of glandular trichome formation was found to be enhanced in LG compared to LF tissues. Besides, the other listed genes (Table 4) might be involved in glandular trichome initiation and development through their activities in epidermal cell fate determination, which warrants further investigations in hop.

The growing body of research suggested that phytohormones play significant roles in trichome initiation, development, and density regulation by mediating downstream genes [53]. Gibberellin and jasmonic acid exhibited a synergistic effect on trichome production, and the effect of these two phytohormones reflected an increased proportion of cells that developed into trichomes, despite an increase in the overall epidermal cell number of the leaves in *Arabidopsis* [54]. The transient exposure to the gaseous hormone ethylene can stimulate the cell division rates and alter the polarity of cells, resulting in an abundance of guard cells and trichomes in cucumber [55]. The zinc finger protein 6 (ZFP6) C2H2 TF gene plays a pivotal role in regulating trichome initiation and patterning by integrating GA and cytokinin signaling in *Arabidopsis* [56]. In our dataset, series of ethylene-responsive and zinc finger genes were differentially expressed in BR and LG tissues (such as ERF4, AP2-like, 1b-like, RAP2-10), which might participate in the LG initiation, development, and density pattern formation in hop. The further study of the involvement of phytohormone-related genes could provide novel insight into the fine-tuning mechanism of their interaction with regulators to dissect the molecular mechanism underlying the organ-specific control of epidermal patterning and the regulatory mechanism of multicellular trichome development in hop.

In conclusion, the present study provides resources for comparative transcriptomics changes in LF, BR, and LG tissues in hop. The tissue-specific differential expression profile provides an insight into the biosynthesis of bioactive compounds associated with primary and specialized metabolism leading to terpenoids, BA, and PF production in hop. Transcriptome analyses and HPLC suggested that the expression of DEGs involved in the terpenoids, BA, and PF metabolic pathways could be correlated with the intensive accumulation of related metabolites in LG tissues in hop. The transcriptome database, candidate unigenes, and resources generated from this study could be useful for future investigation in functional genomics, evolutionary analysis of secondary metabolism in diverged lineage, and closely related species such as *Cannabis* and breeding, and genetic engineering program to enhance the secondary metabolites. Nevertheless, the molecular background and gene regulatory network are restricted to the unicellular non-glandular trichomes in the model species *Arabidopsis thaliana*. The emerging picture suggests that glandular trichomes diverge considerably in terms of morphology, locations, and types of secreted compounds, which further implies that their development is under different transcriptional control across different plant species or even in different trichome types within a single plant species [48]. Our study provides new candidate unigenes, which could be used as a starting point to dissect the gene regulatory network involved in glandular trichome development in hop.

## 4. Material and Methods

### 4.1. Plant Materials

The hop (cultivar Osvald’s 72) plant was grown under standard agronomic conditions at Žatec (Czech Republic). The LF, immature cones (green BR), and matured cones (yellowish-green BRs) samples were collected from the plagiotropic branches in the middle third of the tree on the successive dates in 2017 (LF: 26 July; BR: 10 August; Cones: 5 September). The yellow LGs were separated from the lower part of the dissected BR of mature cones by agitation in liquid nitrogen followed by filtration through 1-mm metal sieve and a 500-μm nylon screen to remove cone debris. The glands were recovered from the liquid nitrogen and stored at −80 °C until RNA extraction. The distribution of glandular and nonglandular trichomes on the LF, green BRs lower and upper parts of the BR/bracteole and in gland preparation were visualized by electron microscope (JEOL JSM-7610F, Jeol, Peabody, MA, USA), which revealed that gland preparation contained a meager amount of nonglandular trichomes without cone tissues (Figure 1). Samples were collected from three biological replicates grown in the same field (coordinates 50.317609, 13.620670).

### 4.2. RNA Extraction, Illumina Sequencing, and Data Processing

Total RNA was extracted from frozen LGs, LF, and BR samples using Concert™ Plant RNA Purification Reagent (Invitrogen, Carlsbad, CA, USA) followed by a cleanup with an RNAeasy Mini RNA kit (Qiagen, Germantown, MD, USA) including removal of DNA contamination using DNA-free^TM^ DNA Removal kit (Thermo Scientific, Waltham, MA, USA) according to the manufacturer’s protocol. In order to maintain uniformity, we used LF and BR samples with an equal number of glands 40 ± 10 cm^−2^. The RNA quantity was determined by the NanoDrop 2000 spectrophotometer (Thermo Scientific, Waltham, MA, USA), whereas the quality and integrity of RNA samples were assessed by Agilent 2100 Bioanalyzer (Agilent, California, CA, USA) using RNA 6000 Nano assay kit (Agilent, USA). The RNA samples with RNA integrity number (RIN) more than 8.0, were used for cDNA preparation. The isolation of mRNA from the total RNA was performed using PolyATract mRNA isolation system IV (Promega Corporation, Madison, WI, USA) and subsequently, 1.5 μg of mRNA was used for cDNA synthesis using Universal Riboclone cDNA synthesis system (Promega Corporation, Madison, WI, USA) following the manufacturer’s protocol. Approximately 600 ng of double-stranded cDNA was sheared via nebulization into small fragments, and fragments ranging in size from 300 to 800 bp were selected for library construction using TruSeq RNA Library Preparation Kits. The quality of libraries was assessed using the Agilent 2100 High Sensitivity DNA kit (Agilent Technologies, Santa Clara, CA, USA). The generated cDNA libraries were paired-end sequenced on an Illumina HiSeq 2500 platform (Illumina, San Diego, CA, USA). The obtained raw sequencing data of LF, BR, and LG were deposited in the NBCI sequence read archive (SRA) with the accession numbers SRX7269218, SRX7225155, and SRX7231776 respectively. The raw sequencing data were subjected to removal of low-quality reads containing primer/adaptor sequences and trimming of read lengths (Phred score < 20) using the Trimmomatic v0.30 program [57]. The quality of reads was evaluated by their error rate, Q20, Q30, and GC-contents using the FASTQC toolkit and reads with length ≥ 45 bp on both sides of the paired-end format were subjected to downstream analysis. The high-quality reads were de novo assembled separately by tissue types (LF, BR, and LG) into unique transcript sequence, termed as unigenes using Trinity software [58] with minimum kmer coverage set to 2 and all other parameters set as default. The assembled unigenes corresponding to LF, BR, and LG was aligned to the hop draft genome assembly [59] using the Spaln2 program [60]. In addition, the unigenes sequences were also compared against the hop transcriptome database of HopBase genomic resources repository (http://hopbase.org/) using MEGABLAST at E-value < 1 × 10^30^, with a cutoff of percentage identity more than 95% and alignment length greater than 100 bp. The transcriptome assembly quality, completeness and accuracy of assembled unigenes was performed using BUSCO (Benchmarking Universal Single-Copy Orthologs; version 3.0) software [61].

### 4.3. Functional Annotation of Hop Assembled Unigenes

To assign putative gene function, unigenes were subjected to BLASTx analysis at E-value cut-off of 1 × 10^−5^ against the NCBI non-redundant (nr) protein database. The BLASTx results were imported to Blast2GO software v2.6.2 [62], and GO terms (http://www.geneontology.org/) with the same E-value for molecular function, biological process, and cellular component categories were assigned to the corresponding hop transcripts. To annotate the protein family information to unigenes, HMMer was used to search against the Pfam database (version 26.0, http://pfam.xfam.org/) using the Pfam_Scan program [63]. The unigenes coding sequence (CDS) and orientation were predicted using BLASTx and ESTScan [64]. In addition, unigenes were searched (BLASTx, E-value cutoff of 1 × 10^−5^) against NCBI COG database (https://www.ncbi.nlm.nih.gov/COG/) and categorized under different functions accordingly. To gain an overview of which pathways are active in LF, BR and LG, unigenes were mapped to KEGG Ortholog database (KAAS; http://www.genome.jp/kegg/kaas/) using the single-directional best hit (SBH) method.

### 4.4. Differential Gene Expression and Biological Pathway Analysis

Reads sequenced from each sample of hop at three different developmental stages were mapped onto the reference transcriptome. To obtain relative expression levels, FPKM value (fragments per kilobase of transcript per million mapped reads) in each sample was counted by expectation-maximization (RSEM) protocol using in-built scripts in the Trinity software package [58]. The data normalization among different libraries was performed using the Trimmed Mean of M-values normalization method in Trinity. The normalized count value was exported to the Bioconductor software package DESeq2 [65] for differential gene expression analysis. The significance of differential gene expression was assessed using the Benjamini–Hochberg false discovery rate (FDR) < 0.05. The expression of a particular gene was considered to be differentially expressed when results from the aforementioned tests were significant at a level of FRD with at least a two-fold change (≥2 or ≤−2) of a ratio of FPKM value of two compared groups. The FPKM value of unigenes was log-transformed and centered, which was used further for calculation of matrix distance for expression heatmap using Euclidean distance and complete linkage methods. A heatmap was constructed using the R statistics package heatmap3 [66]. The GO, COG, and KEGG annotations were performed for the differentially expressed genes (DEGs), as aforementioned. GO terms enriched in DEGs were identified using a hypergeometric test equivalent to one-tailed Fisher’s exact test with an FDR value of 0.05 using the AgriGO toolkit [67] and visualized using ReviGO [68]. The Log_2_FC values of DEGs were assigned to functional categories (or bins) by Mercator software [69] against the *Arabidopsis thaliana* reference database, using the default settings. The mapping file predicted by Mercator was further imported to MapMan software [70] for visualization of the gene expression data obtained from three pairwise comparisons, namely, BR vs. LF, LG vs. BR and LG vs. LF. In the case of expression data for duplicated gene identifiers, the lower value of fold-change was used for the analysis to avoid an overestimation of the data.

### 4.5. Real-Time PCR Analysis of Gene Expression

To confirm the reliability of the results of transcriptome data, qRT-PCR analysis was performed for ten structural, four regulatory genes associated with PF, BA, and flavonoids biosynthesis pathway and two selected candidate DEGs with high or low expression levels using designed specific primers. In addition, differentially expressed structural and regulatory genes associated with PF and flavonoid biosynthesis pathway were subjected for qRT-PCR validation. The extracted total RNA samples of sequencing were used for qRT-PCR analysis. The first-strand cDNA was synthesized by reverse transcription from 5 μg of total RNA in 20 μL of reaction volume using Superscript^®^ III First-strand cDNA Synthesis System (Invitrogen, USA) according to the manufacturer’s instructions. All cDNA samples were 10× diluted and used for qRT-PCR analysis with 200 nM of each forward and reverse gene-specific primers (Table S7) mixed with SYBR Green Real-Time PCR Master Mix (Invitrogen, USA). Additionally, primers specific to hop DEAD-box ATPase-RNA-helicase (DRH) gene were used as an internal control to normalize the variance among samples. The reaction was carried out in 96-well optical reaction plates of qTOWER3 Real-Time Quantitative PCR Thermal Cyclers (Konrad-Zuse-Strasse, Jena, Germany) under the following conditions: 95 °C for 10 min, followed by 40 cycles at 95 °C for 15 s, 58 °C for 30 s and 72 °C for 30 s. The specificity of the PCR primer was examined via melting curve analysis by maintaining the reaction at 95 °C for 1 min, cooling the sample to 55 °C for 1 min and further heating to 95 °C at a rate of 0.5 °C per 6 sec. The relative changes in the expression value of candidate genes were calculated using the comparative Ct (2^−ΔΔCt^) method [71]. The values were represented as the mean of the four biological replicates, each of three biological repeats, and based on that standard error bars were indicated.

### 4.6. Extraction and Quantitative Analysis of Important Secondary Metabolites

The LF, BR, and isolated LG samples were lyophilized prior to the determination and quantification of hop resins (α- and β-acids) and polyphenols (PFs). The lyophilized samples were pulverized into a fine powder and extracted with a mixture of methanol + water (1:1 *v*/*v*) for 12 h at 4 °C. After centrifugation and filtration, using a milling machine (Retsch GmbH, Retsch-Allee, Haan, Germany) the sample was analyzed by high-performance liquid chromatography (HPLC) using a Shimadzu LC-20A (Shimadzu EuropeGmbH, Duisburg, Germany) liquid chromatography with diode array detectors (DAD). The chromatographic separation was performed on a Nucleosil RP C18 (Macherey-Nagel, Düren, Germany, 5 μm, 250 × 4 mm). The sample injection volume was 10 μL and the column temperature was set at 40 °C. The flow rate of the mobile phase was maintained at 0.8 mL min^−1^ in the isocratic regimen. The mobile phase was comprised of a mixture of methanol/water/phosphoric acid (83:16:1). Detection was carried out at wavelengths 314 nm (bitter acids) and 370 nm (PFs). Hop resins, BAs, XN and DMX were quantified by external calibration curves by plotting the areas of peaks against different concentrations of ICE 3, XN and DMX standards (Phytochem, Germany).

## Figures and Tables

**Table 1:** Sequencing statistics for leaf, bracts, lupulin gland.

	Leaf	Bracts	Lupulin Glands	Combined
Raw data				
Reads [M]	66.02	53.15	22.98	127.37
Amount of data [Gb]	7.3	5.9	2.6	12.94
Clean data				
Reads [M]	61.13	50.01	20.18	118.32
Average length [bp]	75.14	108.55	112.25	100.10
Amount of data [Gb]	6.06	5.4	2.2	11.53
Unigenes				
No. of Unigenes (n)	37,690	13,164	7580	43,550
Maximum Length (bp)	19,887	7298	3257	21,367
Average Length (bp)	650	385	396	653
Minimum Length (bp)	105	127	121	144
N25	1743	550	577	1740
N50	1008	398	402	959
GC%	41.5	38.9	39.7	40.9

**Table 2:** Classification statistics for unigenes (UG) and differentially expressed genes (up-regulated (UR) and down-regulated genes (DR)) in leaf vs. bract according to KEGG pathway analysis.

Number of Unigenes in Pathway											
KEGG Categories	Count of Pathways	UG	Bracts	Lupulin Glands	Leaf	Bract vs. Leaf		Lupulin Gland vs. Bract		Lupulin Gland vs. Leaf	
Metabolism						UR	DR	UR	DR	UR	DR
Carbohydrate Metabolism	15	1000	330	222	511	50	160	67	65	23	65
Energy metabolism	07	379	211	152	240	27	79	23	56	4	52
Lipid metabolism	15	581	143	99	310	24	79	33	28	14	27
Nucleotide metabolism	02	205	44	23	106	5	33	5	7	1	13
Amino acid metabolism	14	711	230	155	409	31	115	55	30	18	31
Metabolism of other amino acids	09	202	59	36	112	12	33	9	11	3	12
Glycan biosynthesis and metabolism	11	331	63	23	193	10	18	6	8	0	5
Metabolism of cofactors and vitamins	12	382	105	67	209	15	62	19	22	6	26
Metabolism of terpenoids and polyketides	09	209	56	35	119	15	30	19	23	10	16
Biosynthesis of other secondary metabolites	17	321	95	56	165	40	30	32	20	27	12
Xenobiotics biodegradation and metabolism	18	156	52	35	75	5	19	17	13	4	12
**Genetic information processing**											
Transcription	03	388	95	71	173	16	23	3	10	0	2
Translation	05	835	228	173	505	64	90	12	44	2	18
Folding, sorting and degradation	07	657	182	141	363	35	73	12	29	5	16
Replication and repair	07	469	65	26	281	14	32	5	19	1	8
**Cellular Process**											
Transport and catabolism	09	815	192	129	456	35	100	20	36	4	30
Cell growth and death	11	724	137	79	423	33	68	7	20	2	19
Cellular community—eukaryotes	05	108	23	18	49	7	17	4	4	0	3
Cellular community—prokaryotes	03	60	18	9	30	4	10	2	5	1	4

**Table 3:** Analysis of metabolome content in leaves, bracts and lupulin glands of hop.

	α Acids	β Acids	Cohumulone	Colupulone	XN	DMX	Total Oils
hop sample	% w/w	% w/w	% rel	% rel	% w/w	% w/w	% w/w
lupulin glands	26.06 ± 14.0	17.44 ± 7.8	26.1 ± 4.63	51.4 ± 7.29	1.63 ± 0.78	0.76 ± 0.06	3.24 ± 8.3
bracts/bracteoles *	5.39 ± 0.85	3.44 ± 0.26	23.2 ± 3.87	42.9 ± 5.18	0.39 ± 0.08	0.11 ± 0.03	1.24 ± 0.41
leaves	0.22 ± 0.08	0.16 ± 0.07	ND	ND	0.01 ± 0.00	ND	0.14 ± 0.03

* for this analysis lupulin-enriched part of the bracts was not excised from the sample. The values represent the mean ± standard errors of four different samples (*n* = 4). The samples were taken from three wild-type hop plants (cv. Osvald 72) grown in the field condition in 2017. ND: not detected.

**Table 4:** The list of identified differentially expressed genes and transcriptional factors with their plausible involvement in trichome development.

HopBase ID	Gene/Transcription Factors	Bract vs. Leaf	Lupulin Gland vs. Bract	Lupulin Gland vs. Leaf	Function
HL.SW.v1.0.G039151	*Hl*MIXTA1	NDE	−22.76	NDE	initiation of glandular trichomes
HL.SW.v1.0.G009685	non-specific lipid-transfer protein 1-like	38.87	−3.82	37.49	expansion of epidermal cells and certain secretory tissues.
HL.SW.v1.0.G021650	protein ABIL2	NDE	43.57	27.20	part of a WAVE complex that activates the Arp2/3 complex
HL.SW.v1.0.G041748	signaling peptide TAXIMIN	NDE	53.18	NDE	organ boundary specification between lateral organs and the meristem
HL.SW.v1.0.G010965	xyloglucanase inhibitor 1	NDE	NDE	15.12	involved in the trichome cell wall composition
HL.SW.v1.0.G020081	adaptor protein GIR1-like	4.53	8.59	38.59	involved in the initiation of trichome development; interacts with GLABRA2
HL.SW.v1.0.G008259	R3 Myb ETC1	76.34	NDE	32.51	trichome development repressor
HL.SW.v1.0.G009660	R3 Myb CPC-like	−6.61	NDE	NDE	trichome development repressor
HL.SW.v1.0.G001811	HlMIXTA1 Myb93-like	NDE	62.26	NDE	lateral root development
HL.SW.v1.0.G046443	rax2; Myb 36	−25.8	108.74	NDE	cell differentiation
HL.SW.v1.0.G001378	WRKY4-like	NDE	8.53	NDE	defense response; epidermal cell fate specification
HL.SW.v1.0.G044612	homeobox-leucine zipper protein hox32	18.92	−25.39	NDE	polarity specification
HL.SW.v1.0.G026259	homeobox-leucine zipper protein athb-51	NDE	90.91	42.03	meristem regulator to control leaf or bract morphogenesis
HL.SW.v1.0.G021599	Glabra2	−3.75	NDE	NDE	trichome initiation, trichome spacing
HL.SW.v1.0.G005787	glabrous 11	NDE	48.69	NDE	trichome branching
HL.SW.v1.0.G015009	G2-like family protein; putative transcription factor KAN1	NDE	17.23	NDE	lateral organ polarity
HL.SW.v1.0.G025215	ap2 erf and b3 domain-containing transcription factor rav1-like	4.61	−8.84	NDE	negative regulator of plant growth and development
HL.SW.v1.0.G041557	yabby 1	NDE	−88.54	−16.89	abaxial cell fate determination

NDE = Not differentially expressed.

## Data Availability

The obtained raw sequencing data of leaf, bracts, and lupulin glands were deposited in NBCI sequence read archive (SRA) with the accession numbers SRX7269218, SRX7225155, and SRX7231776 respectively.

## Supplementary Materials

Supplementary materials can be found at https://www.mdpi.com/1422-0067/21/1/233/s1. Figure S1: Correlation indices between different samples; Figure S2: Histogram presentation of clusters of orthologous groups (COGs) classification of unigenes (A) and differentially expressed genes in bract vs. leaf (B), lupulin glands vs. bract (C) and lupulin glands vs. leaf in hop; Figure S3: GO enrichment analysis of differentially expressed genes in bract compared to leaf at a significance level *p* = 0.05, using GO terms from GO Slim; Figure S4: GO enrichment analysis of differentially expressed genes in lupulin glands compared to bract at a significance level *p* = 0.05, using GO terms from GO Slim. GO term enrichment analysis was carried out by Cytoscape software with a Bingo plug-in; Figure S5: GO enrichment analysis of differentially expressed genes in lupulin glands compared to leaf at a significance level *p* = 0.05, using GO terms from GO Slim. GO term enrichment analysis was carried out by Cytoscape software with a Bingo plug-in; Table S1: The unigenes differentially expressed in bract compared to leaf in hop; Table S2: The unigenes differentially expressed in lupulin glands compared to bract in hop. Table S3: The unigenes differentially expressed in lupulin glands compared to leaf in hop; Table S4: Gene ontology (GO) functional enrichment analysis of differentially expressed genes in pairwise comparison of bract vs leaf in hop; Table S5: Gene ontology (GO) functional enrichment analysis of differentially expressed genes in pairwise comparison lupulin glands vs bract in hop; Table S6: Gene ontology (GO) functional enrichment analysis of differentially expressed genes in pairwise comparison of lupulin glands vs leaf in hop; Table S7: The primers used for qRT-PCR.
