# Supplementary material for: Dissection of Dynamic Transcriptome Landscape of Leaf, Bract, and Lupulin Gland in Hop (Humulus lupulus L.)
Source: Int J Mol Sci. 2019 Dec 29;21(1):233. doi: 10.3390/ijms21010233 (PMC6981390; doi:10.3390/ijms21010233)
Supplement: Supplementary file 1 [file ijms-21-00233-s001.zip › ijms-675374--supplementary-final.pdf]

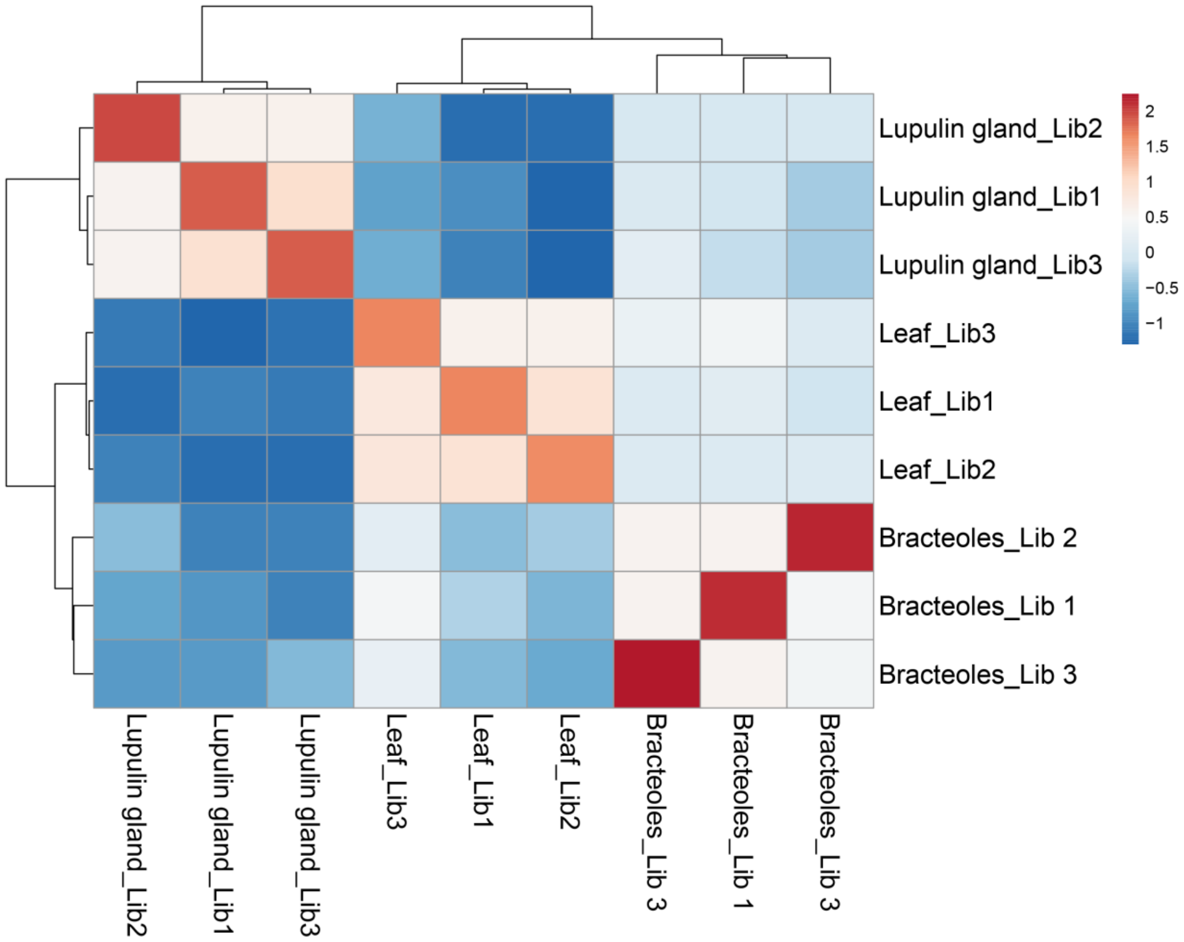

Figure S1: Correlation indices between different samples

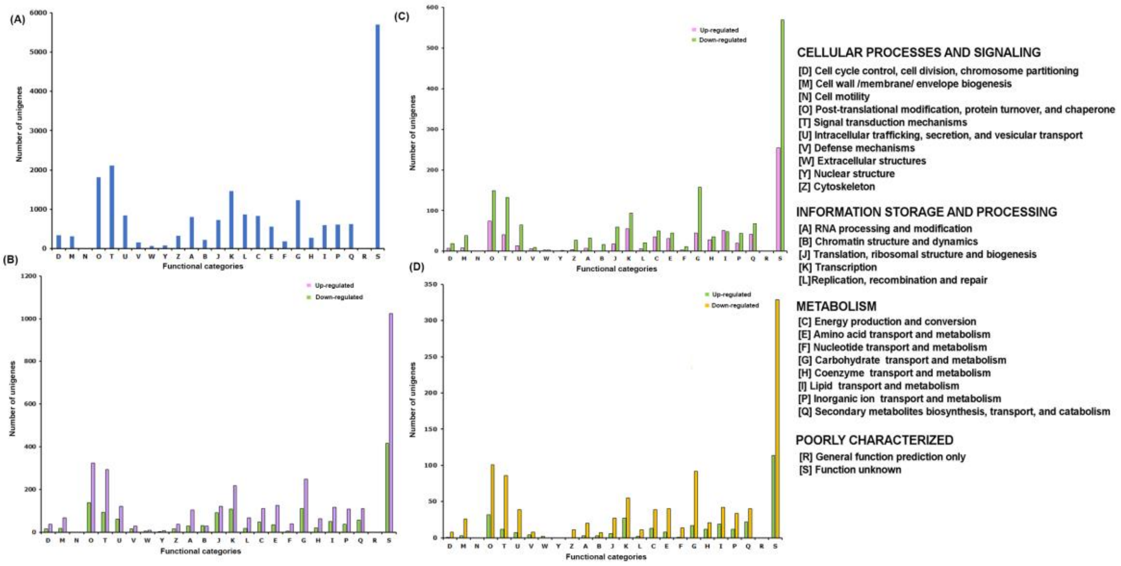

Figure S2. Histogram presentation of clusters of orthologous groups (COGs) classification of unigenes (A) and differentially expressed genes in bract vs leaf (B), lupulin glands vs bract (C) and lupulin glands vs leaf in hop.

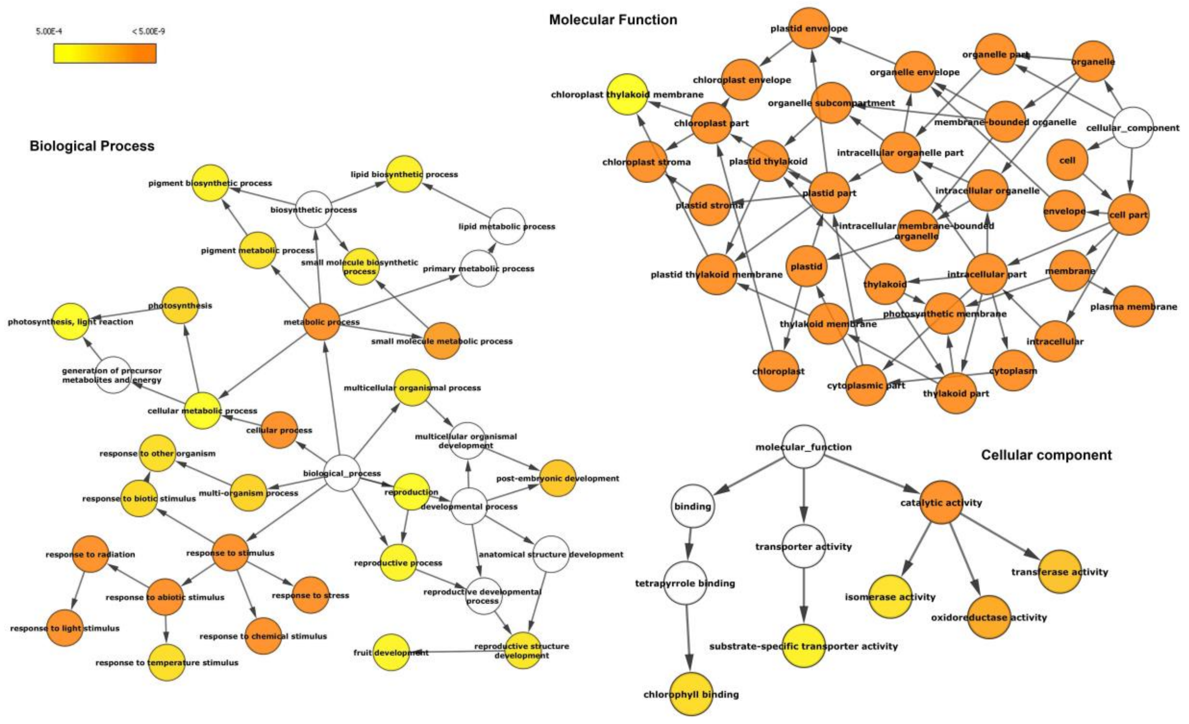

Figure S3. GO enrichment analysis of differentially expressed genes in bract compared to leaf at a significance level p = 0.05, using GO terms from GO Slim. GO term enrichment analysis was carried out by Cytoscape software with a Bingo plug-in. The color of the node represents the (corrected) p-value. White nodes are not significantly over-represented, the other ones are (hypergeometric test, Benjamini & Hochberg False Discovery Rate (FDR) correction), with a color scale ranging from yellow (low) to dark orange (high).

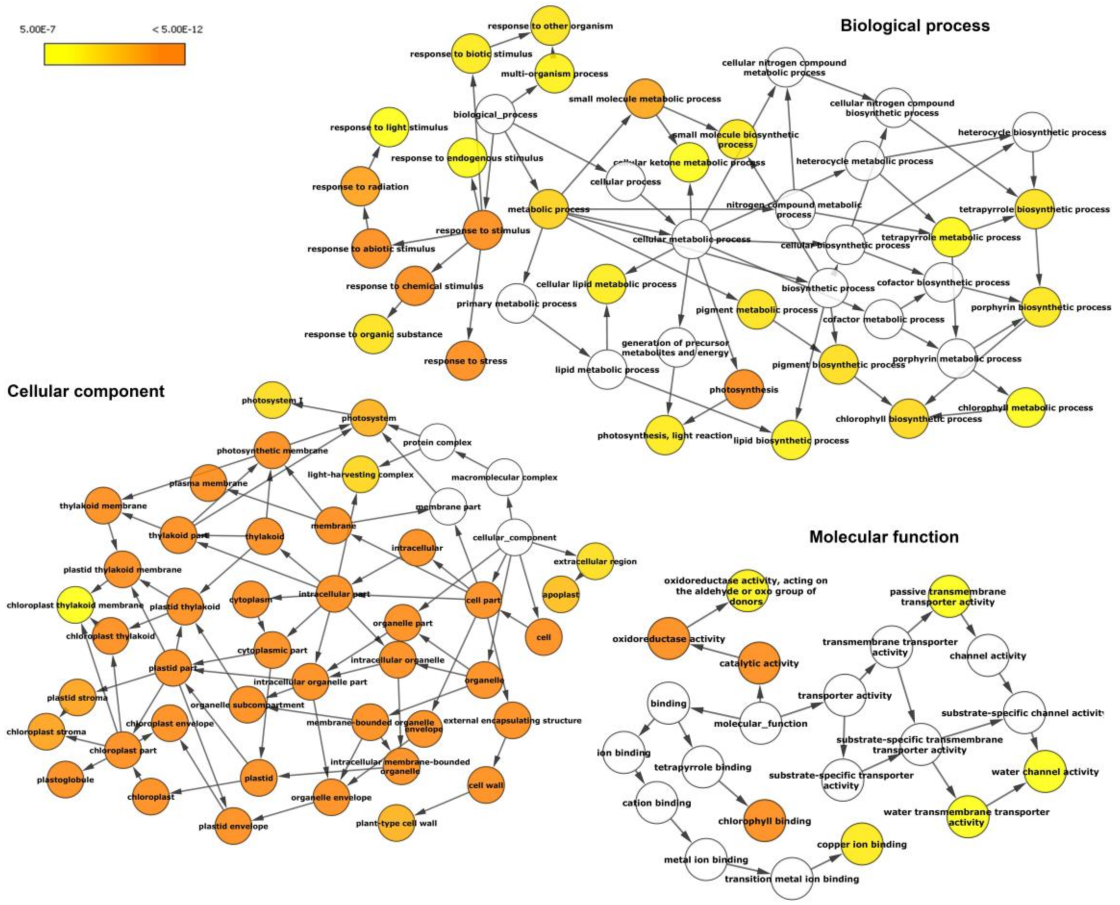

Figure S4. GO enrichment analysis of differentially expressed genes in lupulin glands compared to bract at a significance level p = 0.05, using GO terms from GO Slim. GO term enrichment analysis was carried out by Cytoscape software with a Bingo plug-in. The color of the node represents the (corrected) p-value. White nodes are not significantly over-represented, the other ones are (hypergeometric test, Benjamini & Hochberg False Discovery Rate (FDR) correction), with a color scale ranging from yellow (low) to dark orange (high).

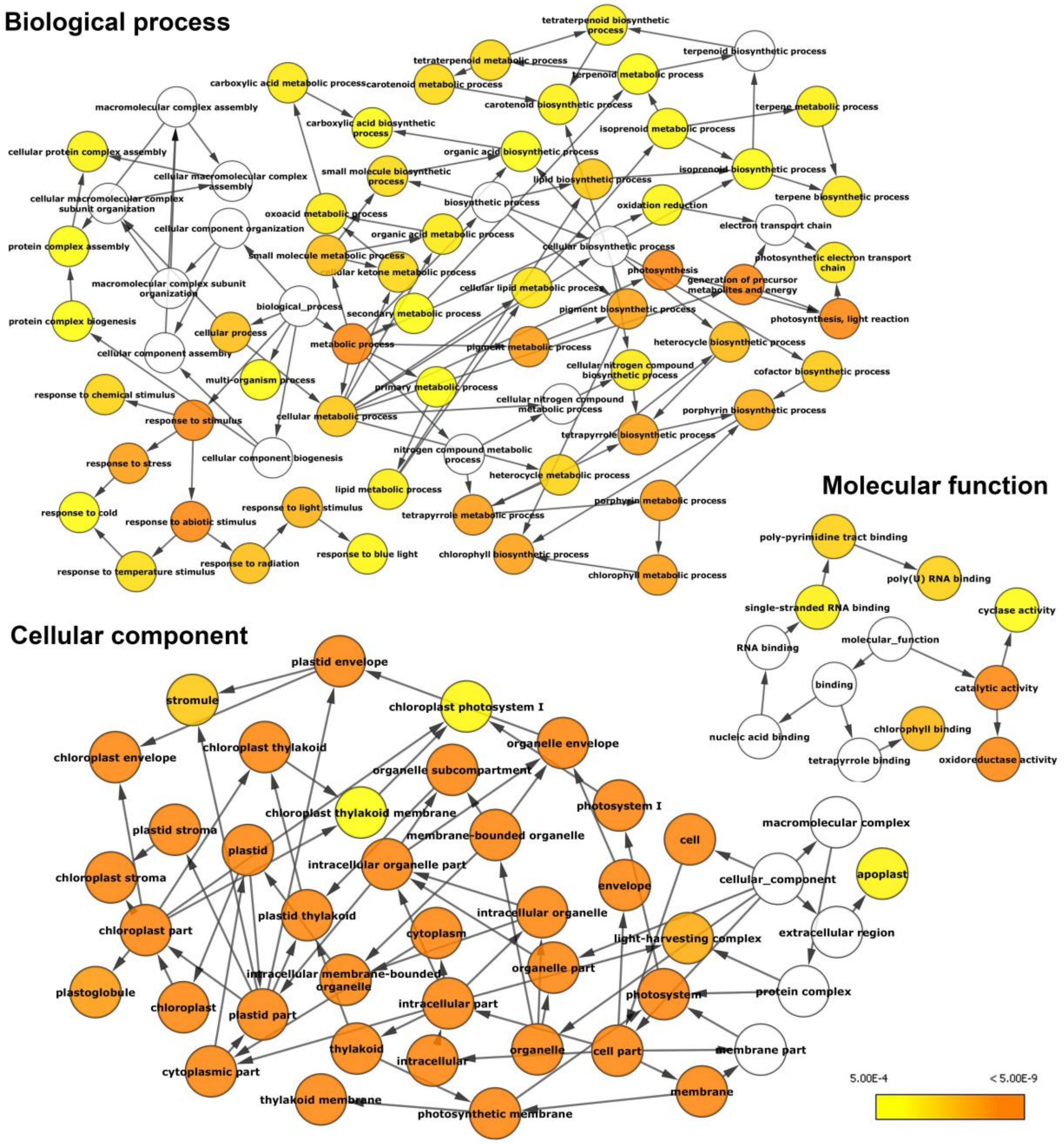

Figure S5. GO enrichment analysis of differentially expressed genes in lupulin glands compared to leaf at a significance level p = 0.05, using GO terms from GO Slim. GO term enrichment analysis was carried out by Cytoscape software with a Bingo plug-in. The color of the node represents the (corrected) p-value. White nodes are not significantly over-represented, the other ones are (hypergeometric test, Benjamini & Hochberg False Discovery Rate (FDR) correction), with a color scale ranging from yellow (low) to dark orange (high).

Table S4. Gene ontology (GO) functional enrichment analysis of differentially expressed genes in pairwise comparison of bracteoles vs leaf in hop.

GO ID	Ontology	Category	Number	Number of	FDR	p-value

			of DEGs	Unigenes in subgroup		
**GO:0008152**	metabolic process	P	510	8180	1.23E-14	6.23E-18
**GO:0044238**	primary metabolic process	P	1276	6595	4.80E-22	8.11E-25
**GO:0005975**	carbohydrate metabolic process	P	168	808	2.12E-03	5.10E-05
**GO:0019538**	protein metabolic process	P	606	3183	9.83E-08	8.80E-10
**GO:0006631**	fatty acid metabolic process	P	22	211	2.49E-02	4.71E-04
**GO:0006629**	lipid metabolic process	P	62	829	1.12E-02	1.86E-04
**GO:0090304**	nucleic acid metabolic process	P	43	1666	8.01E-03	1.21E-04
**GO:0009698**	phenylpropanoid metabolic process	P	20	141	1.75E-03	1.81E-05
**GO:0019748**	secondary metabolic process	P	32	371	3.38E-02	7.15E-04
**GO:0009987**	cellular process	P	590	10275	3.18E-11	4.30E-14
**GO:0006464**	protein modification process	P	349	1942	1.09E-02	3.49E-04
**GO:0009058**	biosynthetic process	P	212	2782	5.03E-11	7.65E-14
**GO:0044550**	secondary metabolite biosynthetic process	P	21	180	9.40E-03	1.46E-04
**GO:0009699**	phenylpropanoid biosynthetic process	P	20	116	1.61E-04	1.33E-06
**GO:0009056**	catabolic process	P	279	1485	4.80E-03	1.33E-04
**GO:0015979**	photosynthesis	P	51	189	5.97E-18	1.01E-21
**GO:0019725**	cellular homeostasis	P	59	303	4.56E-01	4.77E-02
**GO:0009605**	response to external stimulus	P	111	1387	2.44E-06	1.20E-08
**GO:0009725**	response to hormone	P	119	1599	2.89E-05	1.85E-07
**GO:0006952**	defense response	P	77	1145	3.15E-02	6.34E-04
**GO:0051704**	multi-organism process	P	103	1482	2.08E-03	2.18E-05
**GO:0055114**	oxidation-reduction process	P	99	942	5.14E-11	8.69E-14
**GO:0003824**	catalytic activity	F	557	8868	1.43E-17	4.63E-21
**GO:0016740**	transferase activity	F	656	3725	6.95E-04	4.51E-06
**GO:0016836**	hydrolyase activity	F	15	82	5.05E-03	1.48E-05
**GO:0005488**	binding	F	539	10024	7.32E-05	9.51E-08
**GO:0016301**	kinase activity	F	247	1390	2.79E-01	4.88E-03
**GO:0016772**	transferase activity	F	656	3725	6.95E-04	4.51E-06
**GO:0005215**	transporter activity	F	259	1418	8.64E-02	1.07E-03
**GO:0008135**	translation factor activity	F	32	142	7.56E-01	3.04E-02
**GO:0005198**	structural molecule activity	F	124	502	1.33E-04	6.04E-07
**GO:0016853**	isomerase activity	F	28	256	1.12E-02	4.01E-05
**GO:0016491**	oxidoreductase activity	F	63	353	1.00E00	1.60E-01
**GO:0016020**	membrane	C	471	7769	2.36E-12	5.44E-14
**GO:0005737**	cytoplasm	C	818	14521	1.25E-20	2.19E-22
**GO:0005623**	cell	C	1087	22697	1.06E-07	2.83E-09
**GO:0043226**	organelle	C	935	19561	1.29E-03	5.59E-05
**GO:0009536**	plastid	C	450	5292	1.08E-41	4.97E-44
**GO:0005618**	cell wall	C	54	756	2.53E-02	1.38E-03
**GO:0005829**	cytosol	C	140	2290	4.91E-03	2.31E-04
**GO:0005886**	plasma membrane	C	38	262	8.59E-08	2.22E-09

Table S5. Gene ontology (GO) functional enrichment analysis of differentially expressed genes in pairwise comparison lupulin glands vs bracteoles in hop.

GO ID	Ontology	Category	Number of DEGs	Number of Unigenes in subgroup	FDR	p-value
**GO:0008152**	metabolic process	P	848	8180	4.12E-20	2.09E-23
**GO:0044238**	primary metabolic process	P	196	2714	1.00E+00	4.47E-01
**GO:0005975**	carbohydrate metabolic process	P	98	808	1.82E-03	3.14E-05
**GO:0019538**	protein metabolic process	P	273	3183	5.40E-01	5.24E-02
**GO:0006631**	fatty acid metabolic process	P	37	211	1.12E-03	1.65E-05
**GO:0006629**	lipid metabolic process	P	110	829	2.66E-05	1.94E-07
**GO:0090304**	nucleic acid metabolic process	P	91	1666	2.82E-02	9.33E-04
**GO:0009698**	phenylpropanoid metabolic process	P	30	141	3.08E-04	3.59E-06
**GO:0019748**	secondary metabolic process	P	53	371	2.75E-03	5.48E-05
**GO:0009987**	cellular process	P	1003	10275	1.07E-17	9.02E-21
**GO:0006464**	protein modification process	P	154	1942	1.00E+00	6.27E-01

**GO:0009058**	biosynthetic process	P	357	2782	2.75E-17	2.79E-20
**GO:0044550**	secondary metabolite biosynthetic process	P	32	180	3.21E-03	6.56E-05
**GO:0009699**	phenylpropanoid biosynthetic process	P	28	116	1.07E-04	9.22E-07
**GO:0009056**	catabolic process	P	162	1485	1.28E-03	2.04E-05
**GO:0015979**	photosynthesis	P	61	189	4.28E-15	7.23E-18
**GO:0019725**	cellular homeostasis	P	24	303	1.00E+00	8.28E-01
**GO:0009605**	response to external stimulus	P	185	1387	1.78E-09	6.34E-12
**GO:0009725**	response to hormone	P	226	1599	2.72E-14	5.99E-17
**GO:0006952**	defense response	P	139	1145	6.27E-05	5.09E-07
**GO:0051704**	multi-organism process	P	172	1482	4.30E-05	3.42E-07
**GO:0055114**	oxidation-reduction process	P	144	942	7.69E-11	2.47E-13
**GO:0003824**	catalytic activity	F	920	8868	5.38E-23	1.75E-26
**GO:0016740**	transferase activity	F	330	3725	3.27E-01	6.25E-03
**GO:0016836**	hydrolyase activity	F	19	82	1.36E-02	7.94E-05
**GO:0005488**	binding	F	926	10024	4.49E-09	4.38E-12
**GO:0016301**	kinase activity	F	111	1390	1.00E+00	6.42E-01
**GO:0005215**	transporter activity	F	139	1418	2.91E-01	5.20E-03
**GO:0008135**	translation factor activity	F	9	142	1.00E+00	7.50E-01
**GO:0005198**	structural molecule activity	F	63	502	4.33E-02	3.51E-04
**GO:0016853**	isomerase activity	F	33	256	3.46E-01	7.53E-03
**GO:0016491**	oxidoreductase activity	F	219	1492	8.40E-15	5.46E-18
**GO:0016020**	membrane	C	833	7769	2.07E-25	2.86E-27
**GO:0005737**	cytoplasm	C	1340	14521	5.10E-21	8.94E-23
**GO:0005623**	cell	C	1865	22697	4.69E-13	1.21E-14
**GO:0043226**	organelle	C	631	5381	3.69E-26	3.40E-28
**GO:0009536**	plastid	C	635	5292	2.22E-28	6.15E-31
**GO:0005618**	cell wall	C	129	756	1.67E-13	4.01E-15
**GO:0005829**	cytosol	C	245	2290	9.06E-06	4.01E-07
**GO:0005886**	plasma membrane	C	387	3572	4.13E-10	1.26E-11

Table S6. Gene ontology (GO) functional enrichment analysis of differentially expressed genes in pairwise comparison of lupulin glands vs leaf in hop.

GO ID	Ontology	Category	Number of DEGs	Number of Unigenes in subgroup	FDR	p-value
**GO:0008152**	metabolic process	P	510	8180	1.23E-14	6.23E-18
**GO:0044238**	primary metabolic process	P	333	6595	2.49E-01	1.04E-02
**GO:0005975**	carbohydrate metabolic process	P	55	808	1.13E-01	3.43E-03
**GO:0019538**	protein metabolic process	P	142	3183	1.00E+00	9.64E-01
GO:0006631	fatty acid metabolic process	P	22	211	2.49E-02	4.71E-04
GO:0006629	lipid metabolic process	P	62	829	1.12E-02	1.86E-04
GO:0090304	nucleic acid metabolic process	P	43	1666	8.01E-03	1.21E-04
GO:0009698	phenylpropanoid metabolic process	P	20	141	1.75E-03	1.81E-05
**GO:0019748**	secondary metabolic process	P	32	371	3.38E-02	7.15E-04
**GO:0009987**	cellular process	P	590	10275	3.18E-11	4.30E-14
**GO:0006464**	protein modification process	P	77	1942	1.00E+00	3.31E-01
**GO:0009058**	biosynthetic process	P	212	2782	5.03E-11	7.65E-14
**GO:0044550**	secondary metabolite biosynthetic process	P	21	180	9.40E-03	1.46E-04
**GO:0009699**	phenylpropanoid biosynthetic process	P	20	116	1.61E-04	1.33E-06
**GO:0009056**	catabolic process	P	20	182	2.90E-02	5.69E-04
**GO:0015979**	photosynthesis	P	51	189	5.97E-18	1.01E-21
**GO:0019725**	cellular homeostasis	P	13	99	4.35E-02	9.71E-04
**GO:0009605**	response to external stimulus	P	390	5729	4.10E-15	1.39E-18
GO:0009725	response to hormone	P	119	1599	2.89E-05	1.85E-07
**GO:0006952**	defense response	P	77	1145	3.15E-02	6.34E-04
**GO:0051704**	multi-organism process	P	103	1482	2.08E-03	2.18E-05
**GO:0055114**	oxidation-reduction process	P	99	942	5.14E-11	8.69E-14
**GO:0003824**	catalytic activity	F	557	8868	1.43E-17	4.63E-21
**GO:0016740**	transferase activity	F	183	3725	1.00E+00	1.60E-01

**GO:0016836**	hydrolyase activity	F	15	82	5.05E-03	1.48E-05
**GO:0005488**	binding	F	539	10024	7.32E-05	9.51E-08
**GO:0016301**	kinase activity	F	63	1390	1.00E+00	8.42E-01
**GO:0016772**	transferase activity	F	183	3725	1.00E+00	1.60E-01
**GO:0005215**	transporter activity	F	62	1418	1.00E+00	9.47E-01
**GO:0008135**	translation factor activity	F	6	142	1.00E+00	1.00E+00
**GO:0005198**	structural molecule activity	F	22	502	1.00E+00	1.00E+00
**GO:0016853**	isomerase activity	F	28	256	1.12E-02	4.01E-05
**GO:0016491**	oxidoreductase activity	F	143	1492	2.93E-13	1.90E-16
**GO:0016020**	membrane	C	471	7769	1.36	2.36E-12
**GO:0005737**	cytoplasm	C	818	14521	1.27	1.25E-20
**GO:0005623**	cell	C	1087	22697	1.08	1.06E-07
**GO:0043226**	organelle	C	935	19561	1.07	1.29E-03
**GO:0009536**	plastid	C	450	5292	1.91	1.08E-41
**GO:0005618**	cell wall	C	54	756	1.61	2.53E-02
**GO:0005829**	cytosol	C	140	2290	1.37	4.91E-03
**GO:0005886**	plasma membrane	C	38	497	1.72	3.85E-02

Table S7 Primers used for qRT-PCR analyses.

S.No	Primer Name	Sequence (5' - 3')
1	PAL-F	CCGAAGTCTTGTCAGCCATT
2	PAL-R	TGGGGTGATGTCCTAAGAGC
3	4CL-F	TCCGATAGCCTTAACGGTTG
4	4CL-R	CCATAGCCCTGTCCAAGTGT
5	C4H-F	CCACTGGAAGAAGCCAGAAG
6	C4H-R	TCTGCACCAAACGTCCAATA
7	CHS_H1-F	TCACCAACAGCGAGCACA
8	CHS_H1-R	GGTGATCTTCGACTTGGGC
9	OMT1-F	TAAAGGAACAGTGGTGGACGTTG
10	OMT1-R	ACCGCATCAGCACTAGGAATTGA
11	PT1-F	ACAACAACAACAACACCTCTAACA
12	PT1-R	CTTGCAGCTGAAAATTGATAAAG
13	VPS-F	ACTCCGATTGGGATTAATGACTG
14	VPS-R	GTACTCGCTCAGCATTTCAC
15	CHS4-F	TCTGGACTAATGCTTCACTTAAT
16	CHS4-R	AATCTCTAATCCCAATTTCCC
17	DFR-F	CGAAGAAGTTGACAGATTTGGG
18	DFR-R	TTGGTTTGATGGGTAAGAGGA
19	CHI_F	CAACTGCCCTCAACTCAA
20	CHI_R	TTTCTTCCTCAAGCCAAC
21	*Hl*bHLH2-F	AGCGGGTTGACTAGTGTG
22	*Hl*bHLH2-R	ACTTGCACCGTTGTCTCTG
23	*Hl*WRKY1-F	AGATTGATCAGAGCTCCGACAGT
24	*Hl*WRKY1-R	CTTCCTCCATCTATAGCCATCATC
25	*Hl*Myb7-F	CCAGCCAGACCAATTGAAGAG
26	*Hl*Myb7-R	CAAGCCCAAGAAATCATAACCA
27	*Hl*Myb8-F	GGATGATCAGCAGAAACGAC
28	*Hl*Myb8-F	CAACACAAAAACGACAACTGG
29	ERT_F	AAGTGGGGGAAATGGGTATC
30	ERT_R	GCAATCTCCGGTGGACTATT
31	SHCT_F	TCGGAAATGTCCTATCCTGTC
32	SHCT_R	CTTTGAACGAAGTGCCTCTACC

33	DRH-F	ATTCCCACTAAGCGTCAAACC
34	DRH-R	ATCTGCTCTAACCTCCTCTGT
